# An Atypical Case of Acute Pulmonary Embolism in a Sri Lankan Patient With Acute Severe Pancreatitis With Heterozygosity for the SPINK 1 Mutation

**DOI:** 10.7759/cureus.73800

**Published:** 2024-11-16

**Authors:** Tilan Aponso, Athri Wanninayake, Ranjith Peiris

**Affiliations:** 1 Gastroenterology and Hepatology, National Hospital of Sri Lanka, Colombo, LKA; 2 Medicine, National Hospital of Sri Lanka, Colombo, LKA

**Keywords:** acute dyspnea, acute pancreatitis, elevated amylase, pulmonary embolism, spink 1 mutation

## Abstract

Acute pancreatitis is a disease characterized by local destruction of the pancreatic gland due to premature activation of pancreatic enzymes within the acinar cells. Tissue damage can activate an inflammatory cascade, which can lead to systemic complications. Although vascular complications are uncommon, they significantly contribute to mortality and morbidity. Pulmonary embolism being an exceptionally rare complication of acute pancreatitis is reported in only a few cases, and cases of pulmonary embolism without associated deep vein thrombosis in the lower limbs are extraordinarily rare and unreported in the literature.

This article presents the case of a 22-year-old Sri Lankan woman diagnosed with severe acute pancreatitis. On the fifth day of her illness, she developed sudden-onset dyspnea, and imaging revealed a large pulmonary artery embolism affecting the right pulmonary artery and segmental branches in the right lower lobe, with a normal venous duplex scan of the lower limbs.

We started her on anticoagulation and supportive care for her acute severe pancreatitis. Her dyspnea and pancreatitis improved with time. She was later diagnosed as heterozygous for the serine peptidase inhibitor Kazal type 1 (SPINK 1) mutation.

## Introduction

Acute pancreatitis is one of the commonest emergency admissions due to gastroenterological causes in Europe and the USA, with an annual incidence of 13-45/100.000 [1]. In Sri Lanka, alcohol is the most common cause of acute pancreatitis. Other possible causes of acute pancreatitis include biliary pancreatitis, drugs such as corticosteroids and azathioprine, hereditary pancreatitis, autoimmune pancreatitis, hypercalcemia, hypertriglyceridemia, and viruses [1]. Hereditary pancreatitis due to serine peptidase inhibitor Kazal type 1 (SPINK 1) mutation is rare. Around 2% of the general population carries SPINK1 mutations, but less than 1% of those carriers go on to develop pancreatitis [1,2]

Thrombotic vascular complications are rare in acute pancreatitis. Splanchnic venous thrombosis, portal venous thrombosis, and superior mesenteric venous thrombosis are common thrombotic complications in acute pancreatitis, and pulmonary embolism is a very rare thrombotic complication of acute pancreatitis that has less than 10 case reports in the literature. Lower-limb deep vein thrombosis is the main source of pulmonary embolism in all the reported cases. Researchers found that lower-extremity deep vein thrombosis was present in 81.7% of patients with acute pulmonary embolism [3].

We present a case of acute severe pancreatitis with the SPINK 1 N34S mutation. On day five of the illness, the patient had a large pulmonary artery embolism that affected the right pulmonary artery and segmental branches of the right lower lobe. The lower limb did not have deep vein thrombosis. This is the first case in the literature where an acute pancreatitis patient with SPINK 1 N34S mutation had a complicated acute pulmonary embolism without deep vein thrombosis in the lower limb, raising the question of correlation between SPINK 1 and arterial thrombosis.

## Case presentation

A 22-year-old previously well Sri Lankan female patient presented with typical pancreatic pain for two days with reduced urine output. On examination, she was tachycardic with a pulse rate of 110 bpm and a blood pressure of 95/55 mmHg. She had tenderness over the right hypochondrial area without organomegaly.

Diagnostic assessment

She had an amylase level more than five times the upper, low-normal limit. Her creatinine level increased two times from her baseline level (Table 1).

**Table 1:** Investigations

Investigation	On D1-D3	On D14
White cell count	14 x 10^3^	8 x 10^3^
Hemoglobin	11g/dL	11.5g/dL
Platelets	435 x 10^3^	420 x 10^3^
Amylase (40 -100 U/L)	894 U/L	80 U/L
Aspartate aminotransferase (10-35 U/L)	40U/L	42U/L
Alanine transaminase (10-35 U/L)	42 U/L	48U/L
Total bilirubin (0.1 to 1.2 mg/dL)	0.8mg/dL	0.7mg/dL
Alkaline phosphatase (40 to 140 IU/L)	64 IU/L	55 iu/l
Gamma-glutamyl transferase (0 to 30 IU/L)	15 IU/L	18 IU/L
Serum creatinine (0.7 to 1.3 mg/dL)	2.8 mg/dL	0.9 mg/dL
Sodium (135 - 145mEq/L)	137 mEq/L	138 mEq/L
Potassium (3.6 -5.2 mmol/L)	4.3 mmol/L	3.8 mmol/L
C-reactive protein (<0.3 mg/dL)	75mg/dL	10 mg/dL
Procalcitonin (<0.05μg/L)	<0.05μg/L	
Ultrasound scan of the abdomen	Suggestive of acute pancreatitis without local complications Biliary system normal. No gallstones.	
Contrast-enhanced computed tomography of the abdomen		Suggestive of acute pancreatitis without local complications
Magnetic resonance cholangiopancreatography		Normal
Triglyceride level (<150mg/dL)	110mg/dL	
Serum calcium (8.5 -10.2 mg/dL)	9.2mg/dL	
Antinuclear antibody test		Negative
IgG 4 level		Normal
D dimer (<250ng/mL)	3210ng/mL	
Lower limb venous duplex	Normal	
Pulmonary CT angiogram	Extensive pulmonary artery embolism involving the right pulmonary artery and segmental branches of the right lower lobe	
Homocysteine level (<15 micromoles/L)		10 micromoles/L
Antineutrophil cytoplasmic antibodies- cANCA , pANCA		Negative
Genetic studies (MTHFR c.677C>T, Factor V c.1691G>A, and prothrombin)		Negative

She was diagnosed with acute severe pancreatitis, and supportive care was given with intravenous crystalloid fluids and analgesics.

**Figure 1:**
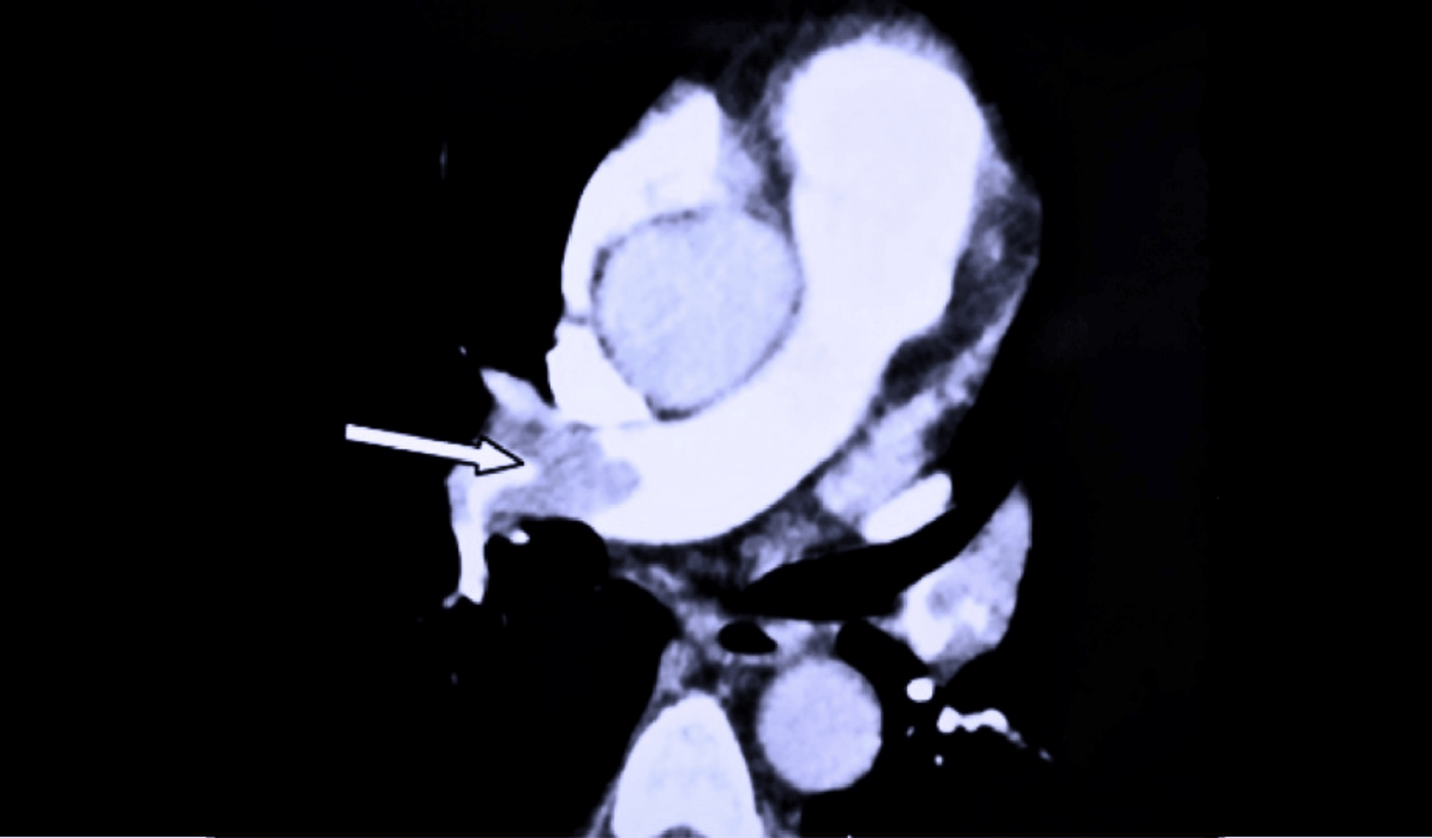
CT pulmonary angiogram: extensive pulmonary artery embolism involving the right pulmonary artery and segmental branches of the right lower lobe

On day three after admission, she developed a sudden onset of shortness of breath with pleuritic-type right-sided chest pain. She was tachypneic and tachycardic with a normal respiratory examination. An urgent electrocardiogram was done, and it showed sinus tachycardia. Chest X-ray and troponin levels were normal, but her D-dimer level was more than ten times higher than the normal value. An urgent pulmonary CT angiogram was performed. It was reported as an extensive pulmonary artery embolism involving the right pulmonary artery and segmental branches of the right lower lobe (Figure 1). There was mild right atrial and ventricular dilatation with grade 1 tricuspid regurgitation and moderate pulmonary hypertension in the transthoracic echocardiogram.

Therapeutic intervention

She was started on subcutaneous enoxaparin, 1.5 mg/kg daily dose, and bridged with Warfarin therapy. Her acute dyspnea was improved with anticoagulation, and her acute kidney injury and pancreatitis were improved with supportive care. She was then started on the Warfarin daily dose according to prothrombin time.

Follow-up and outcomes

Thrombophilia screening and etiology screening for acute pancreatitis were done as an outpatient, and hereditary and acquired causes of thrombophilia were excluded (Table 1).

She underwent genetic studies for pancreatitis because her father was also diagnosed with chronic pancreatitis without a possible etiology. She and her father were found to be heterozygous for the SPINK 1 N34S mutation. The lack of necessary funds prevented genetic testing for her mother and brother, who are asymptomatic. Upon investigating her family history, we discovered that her mother's father suffered from pancreatic cancer.

## Discussion

Acute pancreatitis is associated with inflammation of the exocrine pancreas, causing acinar cell injury. It can trigger a systemic inflammatory response, which progresses into local and systemic complications. The revised Atlanta score defines pancreatitis as mild when there are no local or systemic complications and moderate when there are local or systemic complications without persisting organ failure that resolves within 48 hours. Severe acute pancreatitis refers to the presence of persistent organ failure. Most cases of pancreatitis are mild, accounting for 80% of acute pancreatitis patients who recover without any complications. The remaining 20% of patients had local and systemic complications. The severity of acute pancreatitis influences the mortality rate, which ranges from 3% for mild acute pancreatitis to as high as 20% for acute severe pancreatitis [1,4,5].

**Figure 2:**
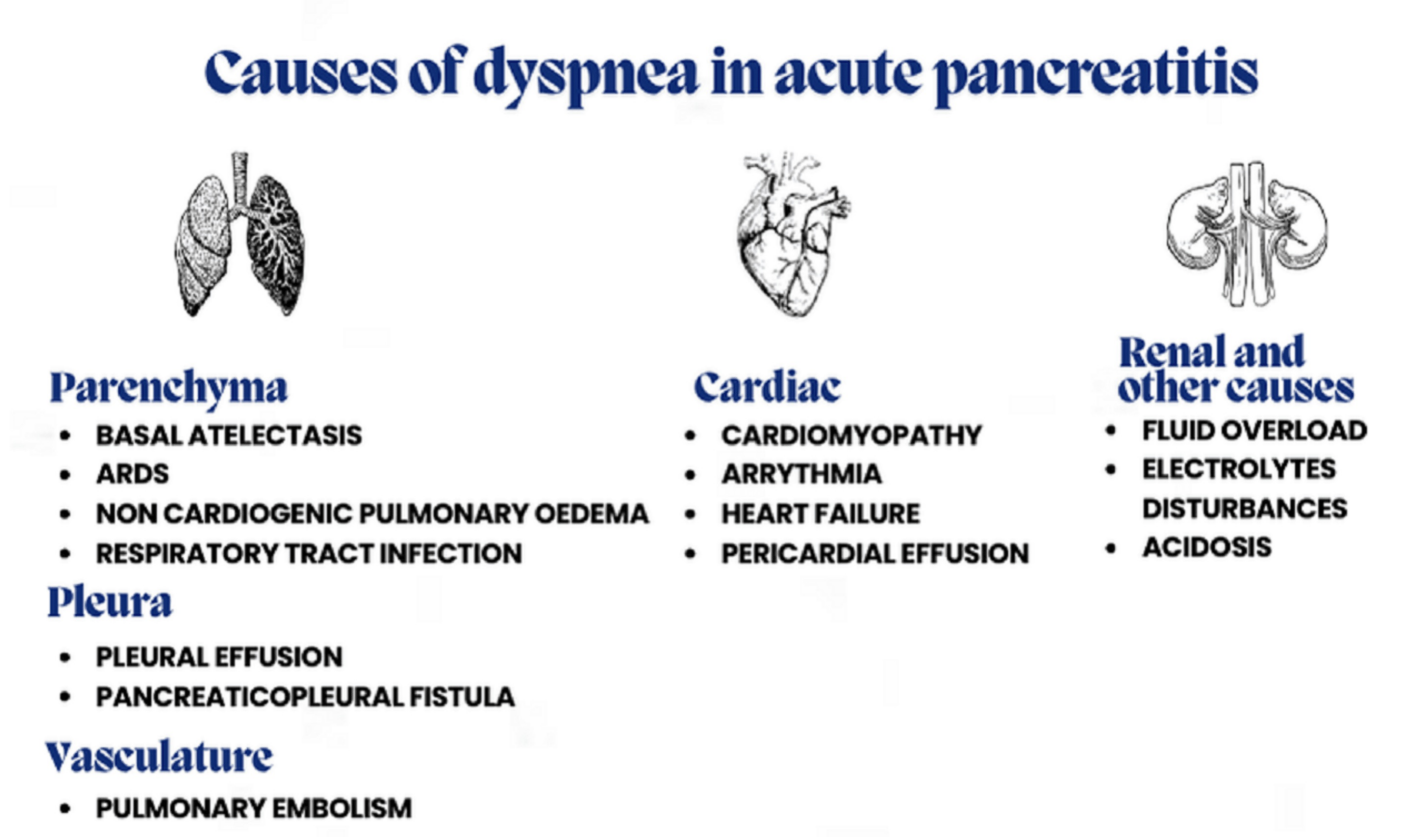
Causes of dyspnea in acute pancreatitis ARDS: Acute respiratory distress syndrome

Patients with moderate and severe pancreatitis may experience local or systemic complications, such as the dyspnea that our patient experienced on day five of acute pancreatitis. This article outlines the most common causes of dyspnea in acute pancreatitis, which we ruled out in our patient (Figure 2).

While evaluating a cause for dyspnea, we diagnosed our patient with acute pulmonary embolism, a condition rarely documented in the acute pancreatitis literature. Pathogenesis for pulmonary embolism following acute pancreatitis is unclear and we proposed that vasculitis and endothelial damage from acute pancreatitis, along with the inflammatory cascade and hypercoagulability caused by high tyrosinemia, could lead to our patient getting a pulmonary embolism.

Venous thrombosis, a rare complication of acute pancreatitis, primarily affects the splenic vein (70%), portal vein, and superior mesenteric vein. The pooled prevalence of splenic vein, portal vein, and mesenteric vein thrombosis was 11.2%, 6.2%, and 2.7%, respectively. However, reports of extrasplanchnic vascular complications are much less frequent. Despite its rarity, it is crucial to rule out pulmonary embolism, as an undiagnosed pulmonary embolism can result in a case fatality rate of up to 20%-30% [6,7].

On etiological evaluation for acute pancreatitis, we found our patient to be heterozygous for the SPINK 1 N34S mutation. Acinar cells in the pancreas secrete serine peptidase inhibitor Kazal type 1, also known as SPINK 1, along with trypsinogen, which interacts with trypsin and inhibits its activity within the acinar cells or the duct. Witt et al. first described the association between the SPINK 1 mutation. Around 2% of the general population carries SPINK1 mutations, but less than 1% of those carriers go on to develop pancreatitis [2]. A review of nine studies with a total of 1493 patients and 2595 controls found a link between SPINK1-N34S in the allelic model and a higher risk of acute pancreatitis, mostly in Caucasians but not in Asians, which also makes this case interesting. The SPINK1 mutation usually acts as a disease modifier rather than causing pancreatitis on its own. It needs additional risk factors for pancreatic damage, like alcohol [1]. In our patient, we excluded all other possible risks for pancreatitis, including biliary stones, alcohol, hypertriglyceridemia, hypercalcemia, and drugs causing pancreatitis. Emerging findings suggest that tumor cells secrete SPINK1, which regulates cancer proliferation, metastasis, drug resistance, transdifferentiation, and cancer stemness [8]. Experts from France discovered that 19 of the 209 carriers of the SPINK1 mutation who had pancreatic symptoms also had thrombosis, suggesting that SPINK 1 may play a role in thrombosis [9]. Our patient, who tested negative for both inherited and acquired thrombophilia but experienced an acute pulmonary embolism, raised the question of whether there is a correlation between SPINK 1 and thrombosis. This is an area that needs to be evaluated in future studies.

## Conclusions

Acute pancreatitis is an important cause of upper abdominal pain, which accounts for 10-40% of the mortality rate. Acute pulmonary embolism is an imperative cause to exclude in a case of dyspnea in an acute pancreatitis patient, which carries a very high mortality rate if missed.
